# Paraplegia from Reflex Causes

**Published:** 1886-10-20

**Authors:** S. T. DeLaMater

**Affiliations:** Fayetteville, N. Y.


### PARAPLEGIA FROM REFLEX CAUSES.

By S. T. DeLaMater, M. D., Fayetteville, N. Y.

The following history of a very interesting case may be of interest to your many readers :

Timothy D——, thirty-two years of age, of Irish parentage, and a man of strong physique and robust health ; he was first seen about one year ago, and at that time gave unmistakable signs of secondary syphillis, for which he received the usual mixed treatment. Gave a history at that time of a chancre six months before. He soon after passed from observation, and was not seen again until January 4 of the present year, when he was found to be suffering from the effects of a long debauch, and gave history of exposure to wet during a hunting expedition. He seemed. at this time, to be suffering from a severe cold, and there was no evidence of paralysis or other lesion. Visited him three times and left him convalescent, but well enough to go out.

January 10—Patient found to be paralyzed from knees downward, and from elbows downward. Anæsthesia was complete over the whole surface affected. Ordered potass iod. grs. x ; hydr-bichlo, gr. ¼ ; three times a day ; also, strych-ferri et phos. acid diluted. The next day tested with electricity. To the Faradaic current there was no response whatever, although in the thighs and other portions of body, except from elbow down, reaction was good ; to the galvanic current the affected muscles responded but very feebly indeed, and only then when thirty cells were used; anæsthesia complete. Iod. increased on the 20th to 30 grs. three times a day, and strych. to grs. 12 t. i. d. Slight improvement shown at this date. This treatment was continued for the next ten days, supplemented by rubbing, bath and occasional use of Faradaic current ; strych. increased to ⅛ gr. t. i. d. at this time. From this time improvement took place steadily in the arms, not much in the legs and feet, and the case seemed to be progressing favorably up to March 5, though subject to occasional relapses. On this date there appeared to be a decided change for the worse in the legs, and the paralysis seemed inclined to extend upward to the thigh. He also complained at this date of trouble in passing water, and urine gave a heavy deposit ; function of the other sphincter not impaired. At this time he was taking potass iod. grs. xxx, t. i. d. ; hydr. bichlor, gr. ¼ ; tried strych. gr. ⅛ ferri et phos. acid.

I was led by this new symptom to examine the penis, and found a degree of phymosis also. Complete circumcision was advised, and performed on the day following, by slitting of the dorsum of the prepuce. The mucous membrane was found to be adhered and much thickened. No anæsthetic was used. Wound healed by granulation as the stitches gave way subsequently. The day of operation he was carefully tested with both currents to the Faradaic ; no contractions whatever, although the strongest battery was used to the galvanic—thirty cell; scarcely any influence in the legs and only slight in the arms.

The second day after operation same tests were made, showing marked improvement in both extremities, and in one week reactions were good in every part to both currents. One month after patient was walking with a cane ; movement of feet and limbs natural ; potass and strych. continued for three months. Patient in good health now.

[One-fourth grain of hydrarg. bichlor, as used above, would be an excessive dose in most cases.—EDITOR RECORD.]

